# Supplementary figures and images for: Paracoccidioides lutzii Infects Galleria mellonella Employing Formamidase as a Virulence Factor
Source: PLoS Negl Trop Dis. 2024 Sep 3;18(9):e0012452. doi: 10.1371/journal.pntd.0012452 (PMC11398694; doi:10.1371/journal.pntd.0012452)

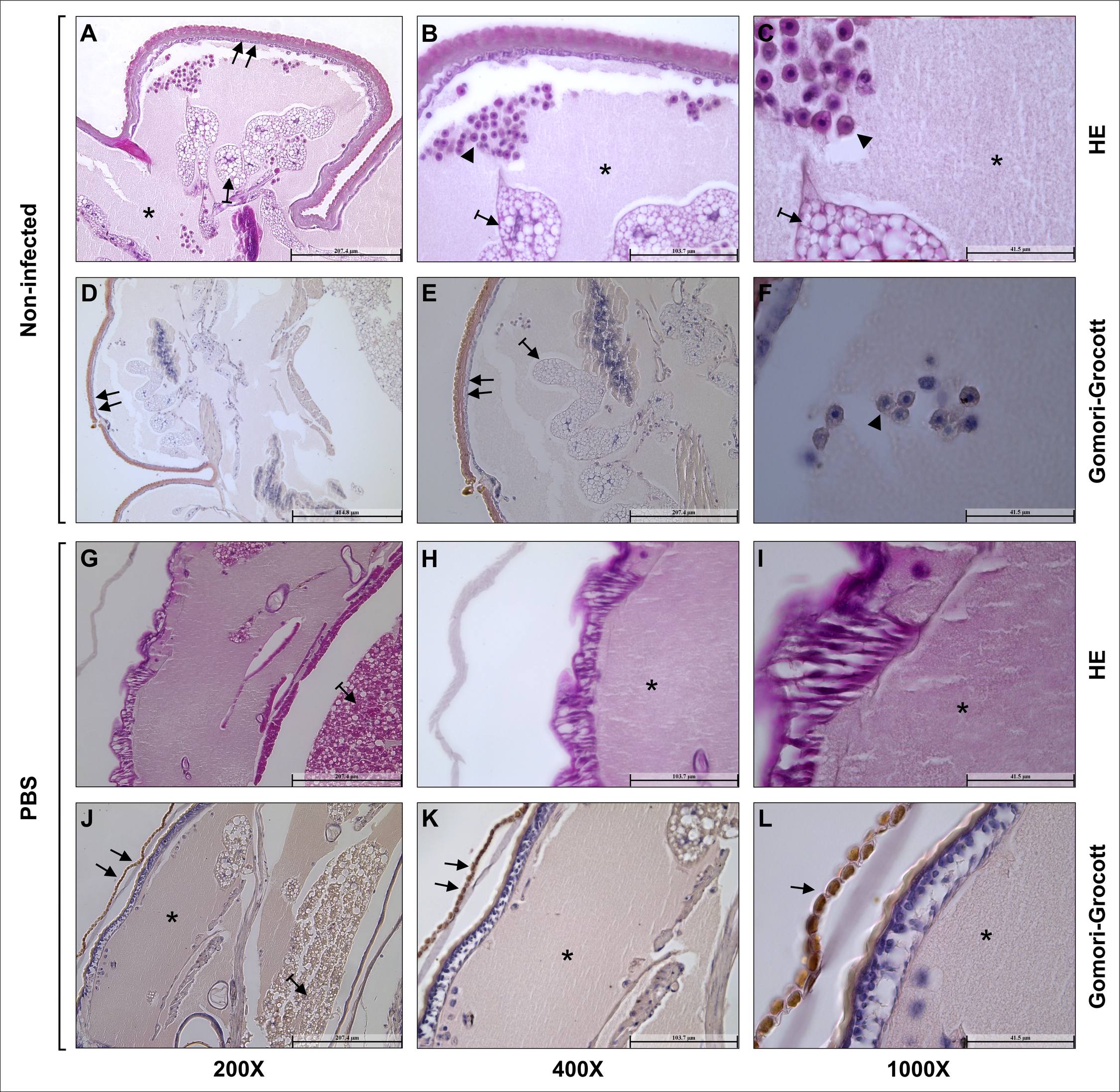

Supplement: S1 Fig — A-C) Tissue slides from non-infected group stained with HE, highlighting Galleria’s cuticle (arrows), peripheral plasmatocytes (arrowhead), adipose cells (dashed arrows), and hemolymph (asterisk). There is no evidence of melanization, nodulation or yeast cells. D-E) Tissue sections stained with silver Gomori-Grocott method confirming absence of yeast structures. The Galleria’s cuticle (arrows), adipose tissues (dashed arrows), and peripheral plasmatocytes (arrowhead) are shown in evidence. G-I) Tissues from the PBS-injected group stained with HE highlights larvae’s hemolymph (asterisk). J-L) Gomori-Grocott stained tissues from the PBS-injected group of larvae. It emphasizes the absence of fungal structures. Galleria’s structures such as cuticle (arrows), adipose tissue (dashed arrow), and hemolymph (asterisk) are apparent. (TIF) [file pntd.0012452.s001.tif]

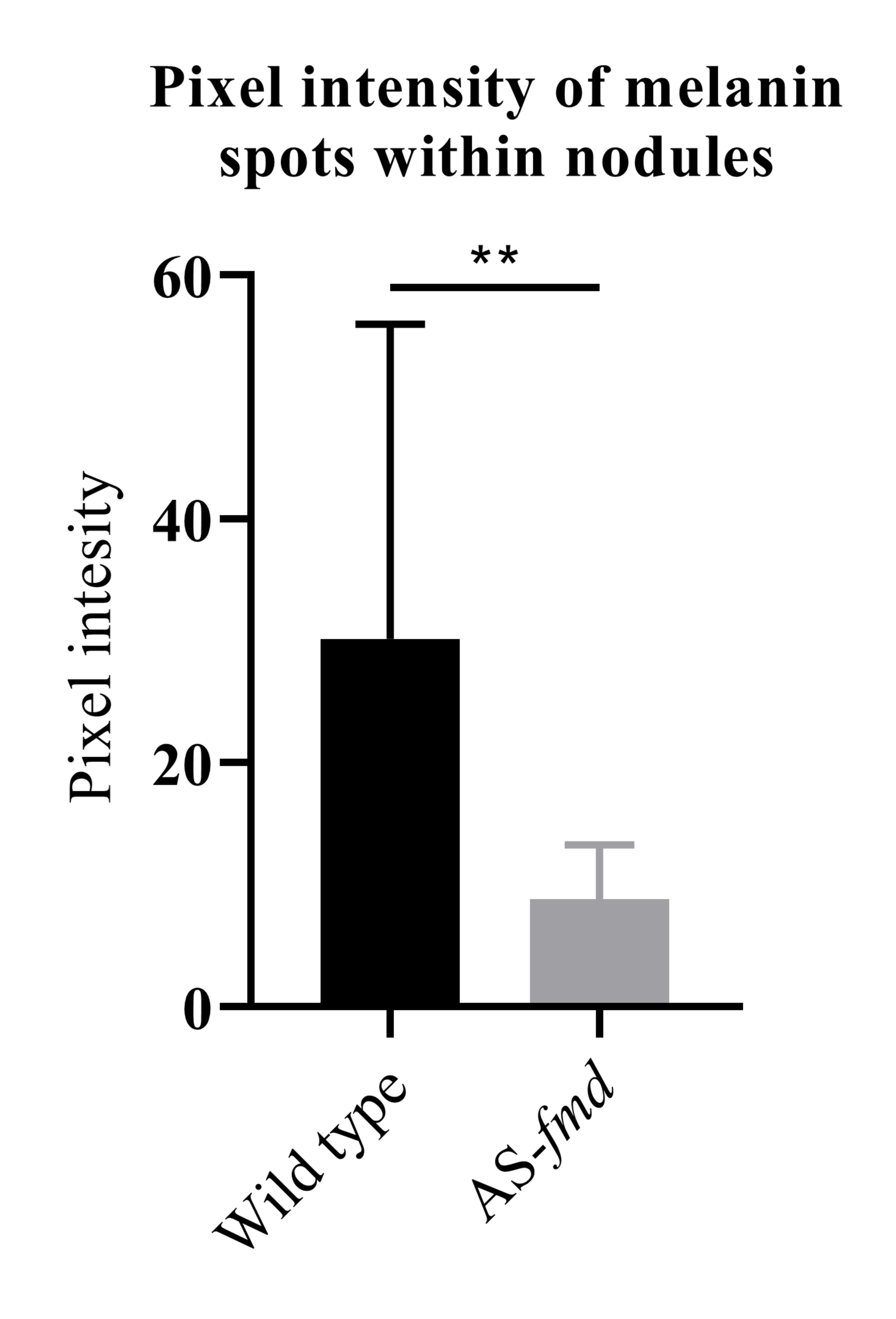

Supplement: S2 Fig — The graphical representation of the pixel measurements obtained from nodular melanin spots of the wild type and AS-fmd strains is presented herewith. The statistical significance of these data was evaluated using a Student’s t-test with the aid of GraphPad Prism8 software. The two asterisks (**) denotate a p-value < 0.001. (TIF) [file pntd.0012452.s002.tif]
