# Supplementary material for: Paracoccidioides lutzii Infects Galleria mellonella Employing Formamidase as a Virulence Factor
Source: PLoS Negl Trop Dis. 2024 Sep 3;18(9):e0012452. doi: 10.1371/journal.pntd.0012452 (PMC11398694; doi:10.1371/journal.pntd.0012452)
Supplement: Attachment — Submitted filename: RESPON~1.PDF [file pntd.0012452.s005.PDF]

UFG

To: Editor and referees of Plos NTD Goiania, July 24th, 2024

Dear Prof. Dr. Josh Nosanchuk,

It is our pleasure to resubmit the revised manuscript (PNTD-D-24-00659) entitled "***Paracoccidioides lutzii*** **Infects** ***Galleria mellonella*** **Employing Formamidase as a Virulence Factor**". We are grateful for the opportunity to respond to the queries posed by the editorial board of the PLOS Neglected Tropical Diseases regarding our manuscript.

The suggestions provided by the reviewers were all highly pertinent, resulting in a significant enhancement in the quality of the manuscript.

The recommendations proposed by the reviewers have been duly considered, and it can be confirmed that the manuscript in question has been revised in accordance with their comments. Additionally, all the images presented on the manuscript were submitted to PACE WebSite (https://pacev2.apexcovantage.com/Upload) and underwent the necessary adjustments.

Please find below a comprehensive list of all questions posed by both reviewers regarding each topic of the manuscript, followed by detailed responses to these inquiries. The primary points raised by each reviewer are indicated by an underlined text.

Reviewer's comments, questions and answers

Reviewer 1 - Material and methods: The objectives of the study are clearly articulated, focusing on the role of *P. lutzii* formamidase during interaction with *G. mellonella*. The study design appears appropriate for addressing the objectives. The use of *G. mellonella* larvae as a model organism to study fungal-host interactions is well-founded. The manuscript provided information regarding the sample size. The authors need to clarify whether appropriate statistical analyses were conducted to support the conclusions, particularly in relation to the results presented in Figure 3. It is essential to specify the statistical methods used, and whether any comparisons between groups were made to validate the findings.

Answer: The goal of the histological analysis presented in Figures 1 to 3 of the manuscript was to assess qualitative parameters related to the discussed indicators of virulence. However, the potential of both *P. lutzii* strains to kill the larvae was quantitatively evaluated through the Kaplan-Meier survival curve

(Figure 4). Furthermore, to ascertain the homogeneity of melanogenesis (Figure 1), nodular structure (Figure 2) and fungal burden (Figure 3), these traits were evaluated qualitatively in both the first and third portions of bodies from two larvae (see Figures 1 to 3 for reviewer's answer).

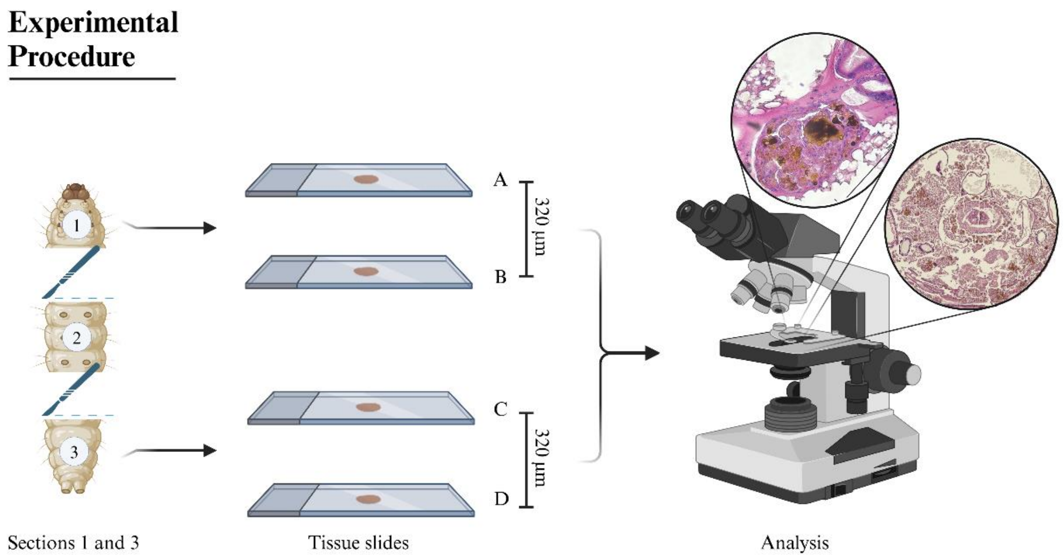

Figure 1 for reviewer's answers – The experimental procedure performed on larvae in order to obtain a histological analysis. Two specimens from each group were sectioned into three portions of similar size. Two tissue slides were prepared from the first (head) and third (tail) sections, with a distance of 320 µm between them. Subsequently, the images were captured and analyzed via microscopy. This figure was created using BioRender.com.

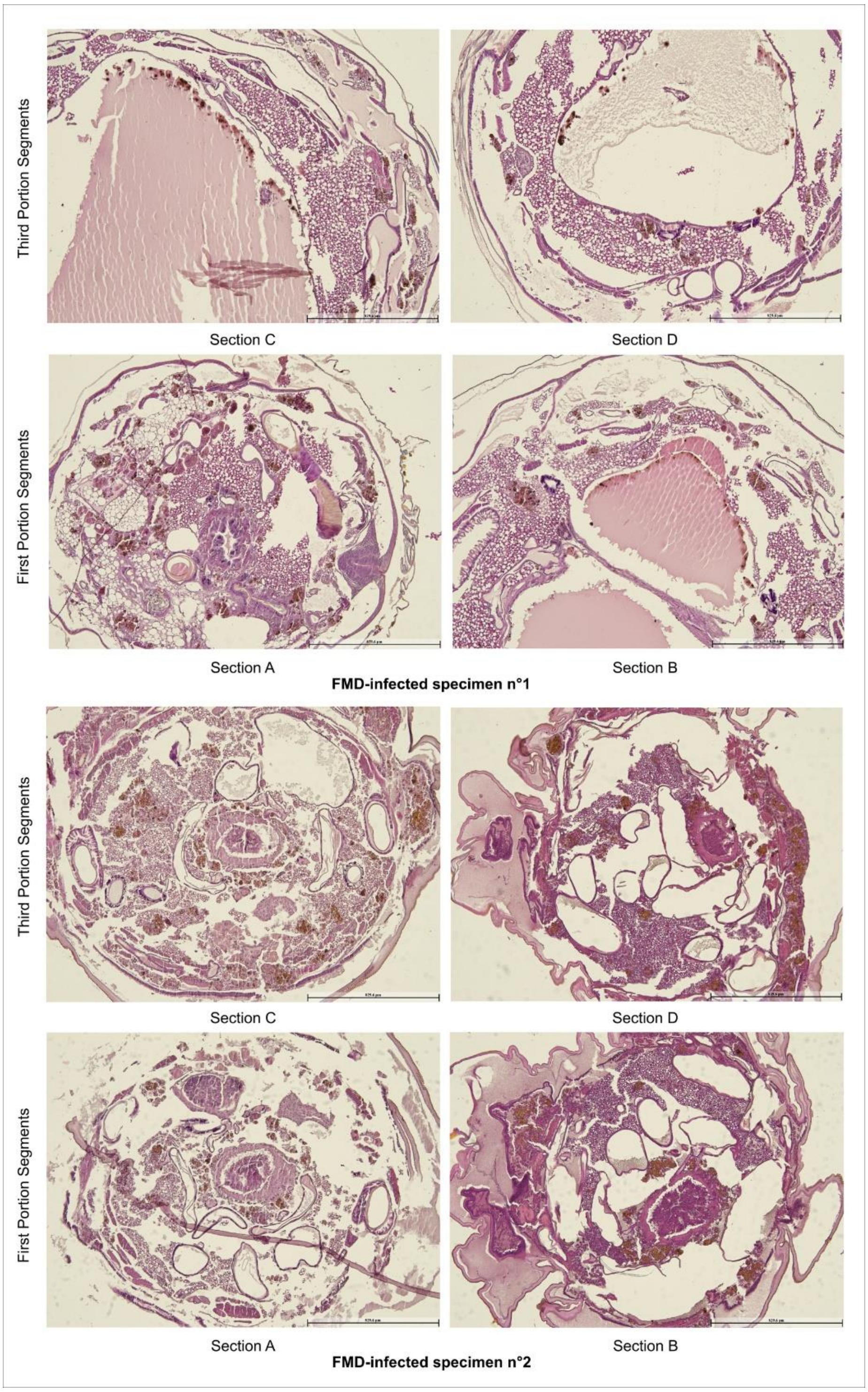

Figure 2 for reviewer's answers – The images displayed are tissue slides from two larvae infected with AS-*fmd*, presenting a pair of sections from both specimens at 50X magnification.

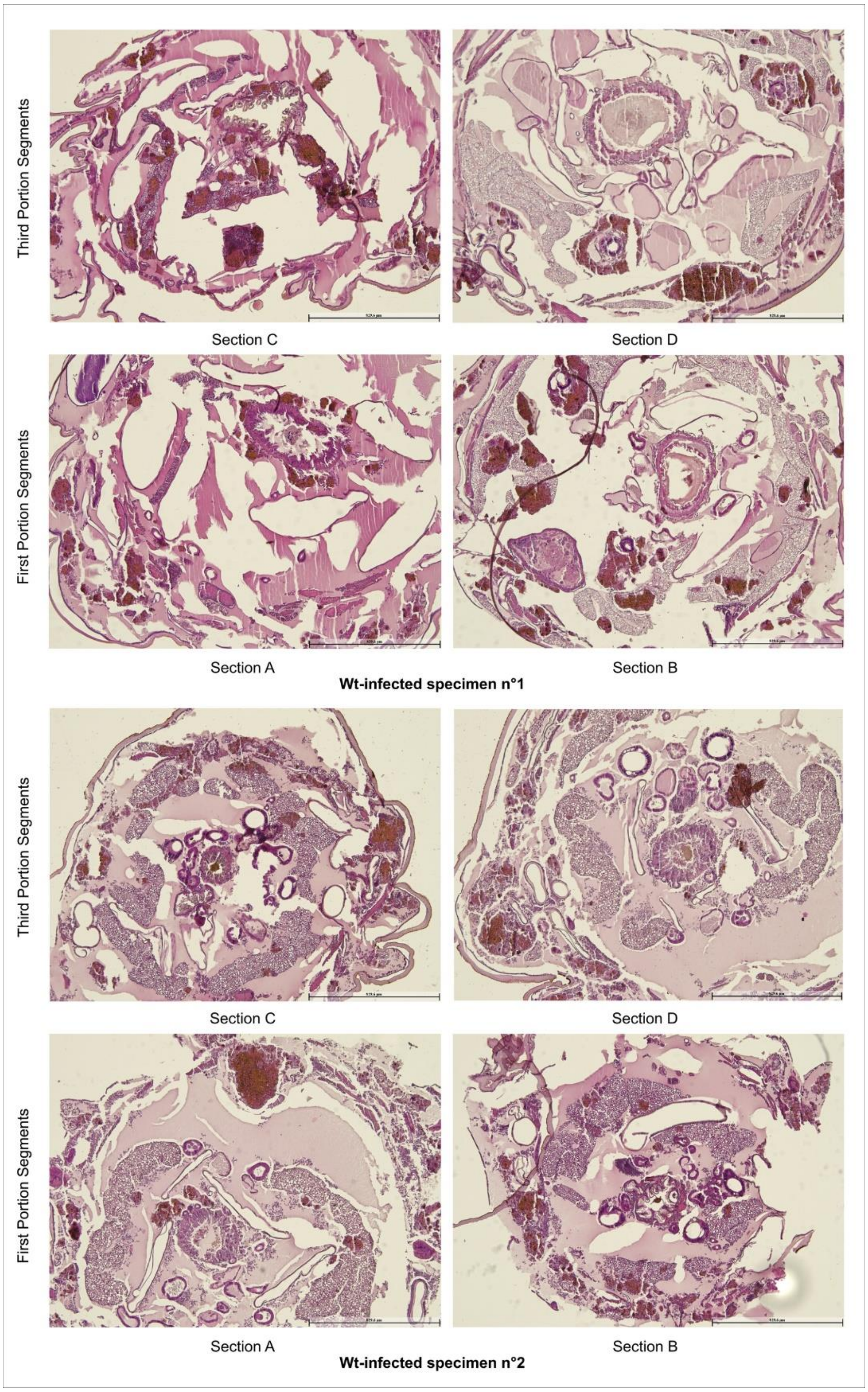

Figure 3 for reviewer's answers – This figure presents the tissue slides from two larvae infected with the wild type *P. lutzii* strain at 50X magnification. Each slide contains a pair of sections from both specimens.

In this analysis, we sought to demonstrate the influence of formamidase reduction on virulence-related characteristics of two larvae by employing histological techniques. Our histological findings in these four specimens revealed discrepancies in larval tissues between the wild type and AS-*fmd* groups. As previously discussed in the manuscript, melanization, nodulation, and fungal load exhibited alterations in the AS-*fmd*-infected group.

Reviewer 2 – Material and methods:

Are the objectives of the study clearly articulated with a clear testable hypothesis stated?

-Is the study design appropriate to address the stated objectives? YES

-Is the population clearly described and appropriate for the hypothesis being tested? YES

-Is the sample size sufficient to ensure adequate power to address the hypothesis being tested? YES

-Were correct statistical analysis used to support conclusions? YES

-Are there concerns about ethical or regulatory requirements being met? YES

Additional comments:

1. Did the authors perform a growth curve of the control and the silenced (AS-find) strain for growth rate control purposes?

Answer: The growth curve and viability of the wild type and AS-*fmd* strains used here were previously assessed over a 96-hour period in brain and heart infusion broth (BHIB) through propidium iodide staining (1). Their findings showed no significant differences in the growth and viability of these two strains. These results demonstrate that the formamidase knockdown process does not impair *P. lutzii* ability of budding and survival under nutrient-rich conditions. Therefore, despite the decrease in formamidase itself, the AS-*fmd* strain should have no other potential reason to be less virulent in larvae of *Galleria mellonella*.

Reviewer 1 - Results: The results are well-presented, but there are points that need more detailed explanations. Additional information is needed regarding the construction of the AS-fmd strain…

Answer: We included the following description about the transformant construct in the section of methods of the manuscript (Page 13 of the manuscript, 'Construction of the AS-*fmd* silenced *P. lutzii* strain').

'The *P. lutzii* formamidase gene (*fmd*) was silenced by the antisense RNA (aRNA) technique in conjunction with *Agrobacterium*-mediated transformation (ATMT), as previously described (1). Briefly, forward (5′ CCGCTCGAGCGGCTTGCATAACCGCTGGCATC 3′) and reverse (5′ GGCGCGCCTCGTCGGCGGAATCGTTATT 3′) oligonucleotides were designed to generate an antisense fragment (AS) of the *P. lutzii* formamidase gene. A binary plasmid (based on pUR5750) containing the AS-*fmd* and a gene for resistance to hygromycin B was then randomly inserted into the

P. lutzii genome. Integration of this transfected cassette into the fungal genome was confirmed by conventional PCR using pUR5750 oligonucleotides.'

Reviewer 1 - Results: …and further exploration of the findings in Figures 1 and 2.

Answer: Thank you for these observations. We included some additional detail in the text, as well as pixel-intensity analyses as supplementary material (Supp. figures 2 and 3). Also, in this work, we did not intend to evaluate detailed mechanisms of immune recognition of formamidase activity/protein and its impact on melanogenesis, nodulation and fungal burden. Rather, we aimed to raise these questions by proposing new hypotheses and creating new knowledge gaps in the field. We are working to continue exploring these gaps to better understand the role of formamidase in this interaction.

Reviewer 2 – Results:
-Does the analysis presented match the analysis plan? YES
-Are the results clearly and completely presented? YES
-Are the figures (Tables, Images) of sufficient quality for clarity? YES
Additional comments:
Fig. 1. Could the authors measure the intensity of the spots and transform it to numeric data?

Answer: Thank you for your recommendation, as suggested, we conducted the measurement of the pixel intensity of melanin spots within the three nodules depicted in Figure 1 of the manuscript, as well as an additional three nodules from the same animals (Figures 4 and 5 for reviewer's comments). The numerical data is presented in Tables 1 and 2 for the reviewer's consideration (see below). Furthermore, the data are presented in graphical form (Figure 6, in response to the reviewer's comments). As anticipated, the pixel intensity was markedly elevated in the nodules of larvae infected with the wild-type strain when compared with the AS-*fmd*-infected strain ($P < 0.001$). Furthermore, we elected to incorporate the tables into the manuscript's supplementary material (Page 17, Supplementary material 3; the two tables were concatenated into only one to the manuscript), accompanied by a graphical representation of the data.

Image/strain	Total area	Mean intensity	Min/Max pixel count	Measured area (%)
**1/Wild type**	2764800	10.9	0/255	4.3
**2/Wild type**	2764800	13.8	0/255	5.4
**3/Wild type**	2764800	78.9	0/255	31.0
**4/Wild type**	2764800	20.8	0/255	8.2

**5/Wild type**	2764800	38.9	0/255	15.7
**6/Wild type**	2764800	17.6	0/255	6.9

Table 1 for reviewer's answers – Table presenting the pixel measurements of melanin spots located within the nodules of wild type-infected larvae. A total of six images underwent the process using the FIJI software.

**Image/strain**	**Total area**	**Mean intensity**	**Min/Max pixel count**	**Measured area (%)**
**1/AS-*fmd***	2764800	13.2	0/255	5.2
**2/AS-*fmd***	2764800	4.4	0/255	1.7
**3/AS-*fmd***	2764800	6.3	0/255	2.5
**4/AS-*fmd***	2764800	15.1	0/255	5.9
**5/AS-*fmd***	2764800	8.9	0/255	3.5
**6/AS-*fmd***	2764800	5.1	0/255	2.0

Table 2 for reviewer's answers – Table presenting the pixel measurements of melanin spots located within the nodules of AS-*fmd*-infected larvae. A total of six images underwent the process using the FIJI software.

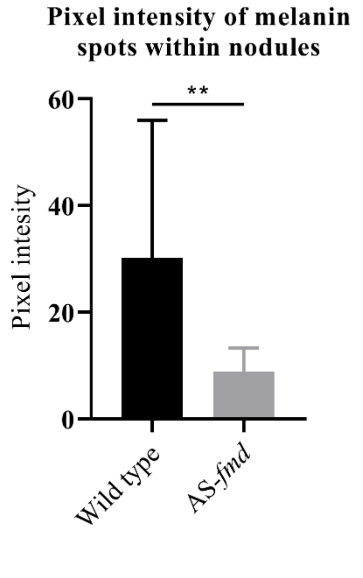

Figure 4 for reviewer's answers – The graphical representation of the pixel measurements obtained from nodular melanin spots of the wild type and AS-*fmd* strains is presented herewith. The statistical significance of these data was evaluated using a Student's t-test with the aid of GraphPad Prism8 software.

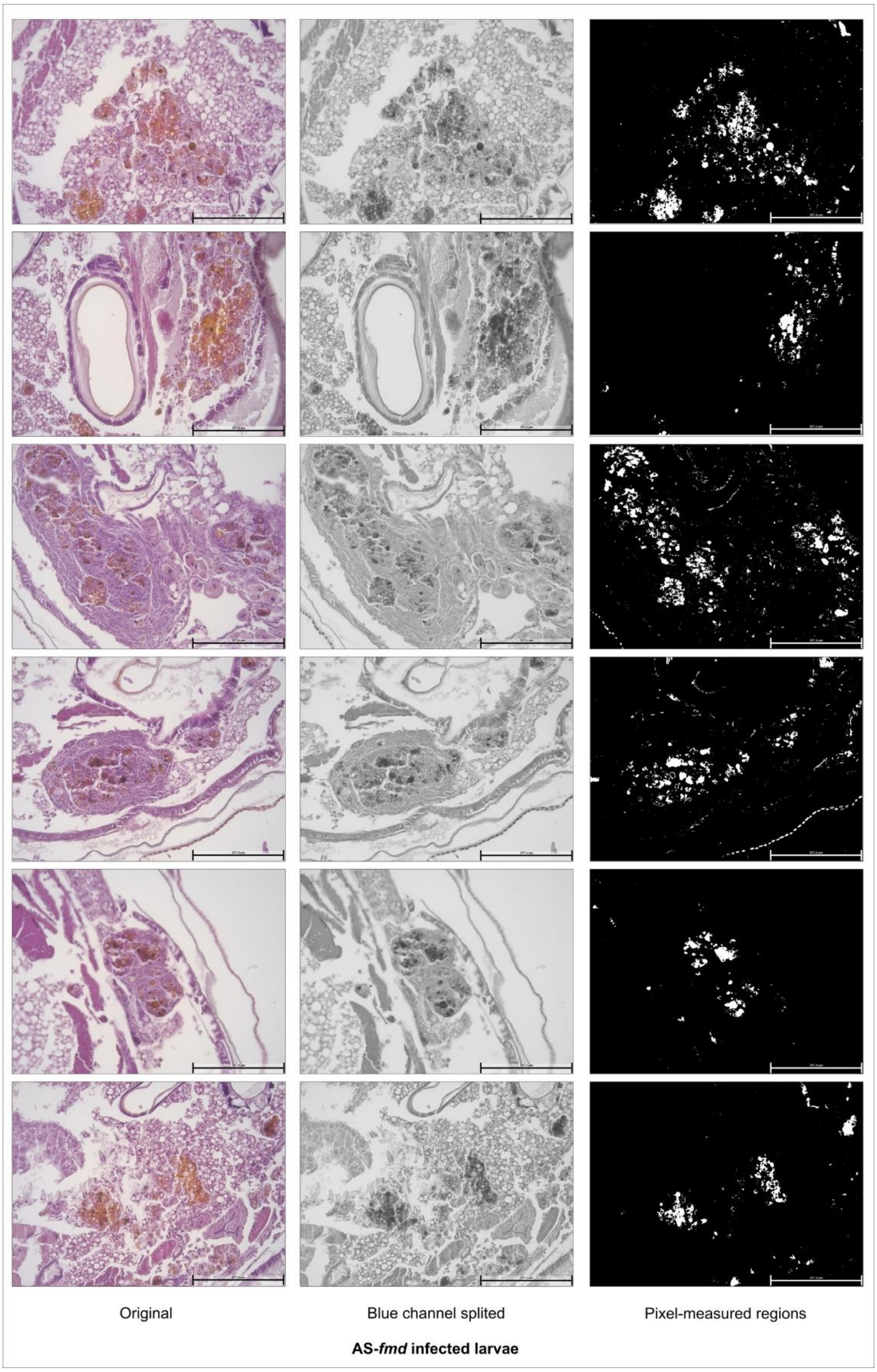

Figure 5 for reviewer's answers – The images displayed are captured from the tissue slides of the AS-*fmd*-infected larvae. They include the original images, the blue channel images, and images that highlight the regions where pixel measurement was conducted, specifically in areas identified as melanin spots (in white).

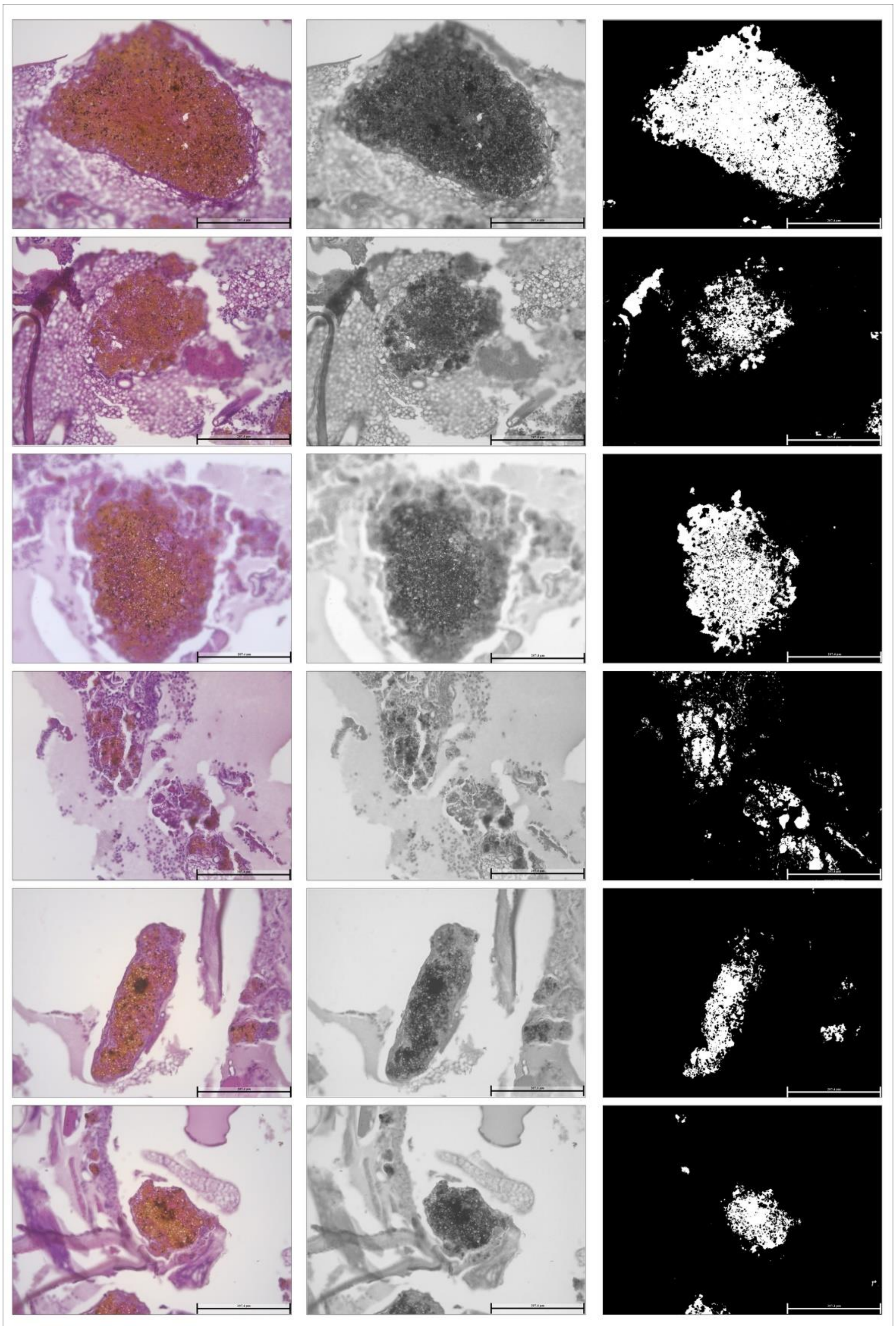

Figure 6 for reviewer's answers – The images displayed are those captured from the tissue slides of the wild-type-infected larvae. They include the original images, the blue channel images, and images that highlight the regions where pixel measurement was conducted, specifically in areas identified as melanin spots (in white).

Reviewer 2 – Results:

Also, is the <u>formation of these melanin spots correlated to the viability of the fungus</u>?

Answer: Thank you for this observation. No, the melanin spots are not correlated with fungus viability. Since the viability of the strain was evaluated after 48 hours of cultivation and exhibited no notable decline, we conclude that the melanization patterns are due to the formamidase expression gap between the strains.

What happens if the authors inject a non-viable wild type to *G. mellonella*?

Answer: It is a trick question and needs to be investigated. The presence of formamidase in the cytoplasm and cell wall, even following cell death, may serve as part of the larval innate immune response trigger, contributing to the dynamics of molecules present in living as well as viable cells. Conversely, the secreted formamidase action will be absent in non-viable cells. We are engaged in the construction of a new recombinant formamidase, as well as the generation of specific anti-formamidase antibodies that will be employed to assess this intriguing question in future investigations.

Reviewer 1 – Conclusions: The conclusions are supported by the presented data, but it would be interesting to explore the hypotheses highlighted in Figure 5 further to ensure they are fully substantiated by the results. The authors should address the public health relevance in detail.

Answer: In light of the suggestions, we elected to modify Figure 5 of the manuscript, withdrawing the bottom image of phagocytosis. This decision is due to the fact that our results indicate the possibility of this occurring, although we are not yet in a position to provide definitive proof. The initial and revised versions of the figure are presented below for your reference. The image at the bottom of the figure, which depicts the process of phagocytosis, has been removed due to concerns regarding the experimental procedures employed. Furthermore, the alteration of terminology from "formamidase activity" to "formamidase" resulted in a more accurate representation of the data in the figure, aligning it more closely with the text. This modification clarifies to the reader that we have not yet determined whether the primary immune trigger is the protein or the enzymatic activity.

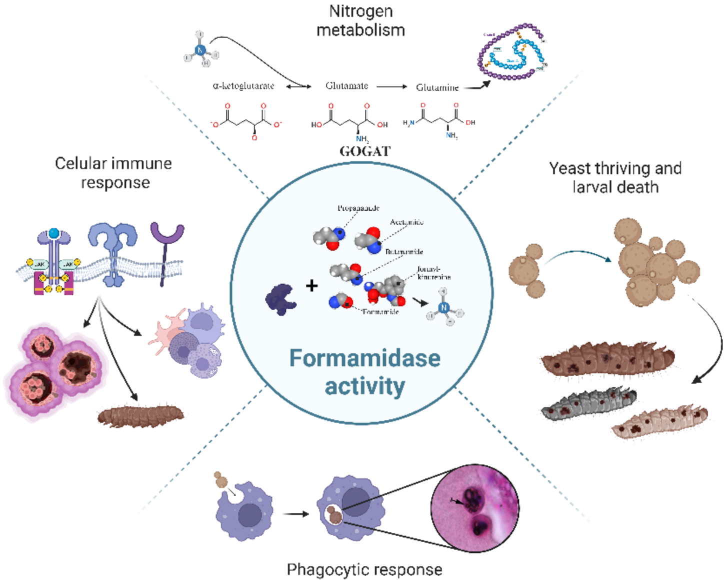

Figure 7 for reviewer' answers – First version of the figure 5 from the manuscript.

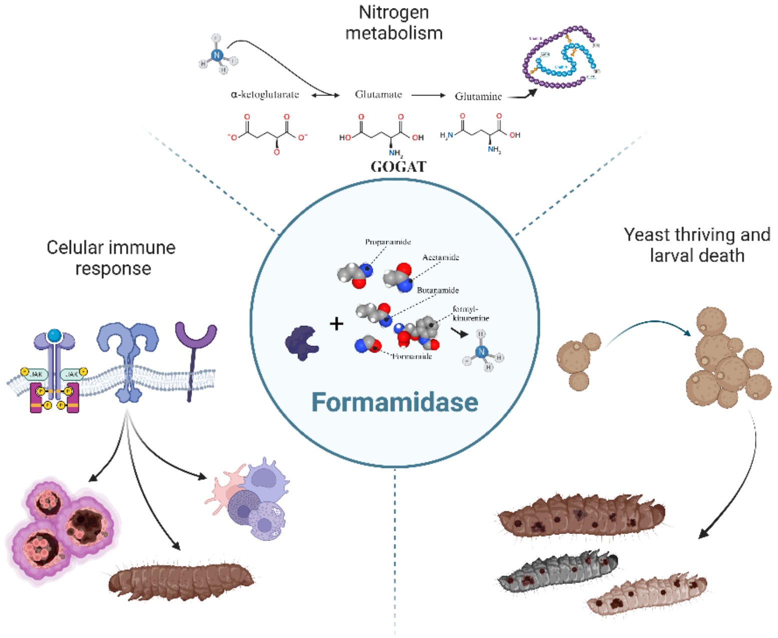

Figure 8 for reviewer' answers – Updated version of Figure 5 from the manuscript. This is the revised version of Figure 5, which replaces the original figure in the manuscript. The revised version excludes the phagocytic response and replaces the central term, "Formamidase activity," with "Formamidase."

Reviewer 2: -Are the conclusions supported by the data presented? YES
-Are the limitations of analysis clearly described? YES
-Do the authors discuss how these data can be helpful to advance our understanding of the topic under

study? YES
-Is public health relevance addressed? YES

SUMMARY AND GENERAL COMMENTS

Reviewer 1: Pereira et al. explore the role of *P. lutzii* formamidase during interaction with *G. mellonella* larvae. The experiments were well-executed, but some concerns need to be addressed:

1. Revise the reference formatting to correct inconsistencies throughout the manuscript.

Answer: We found out those formatting issues and corrected them to match the Vancouver citation style.

2. Include additional information regarding the construction of the AS-*fmd* strain in the methods and/or results sections.

Answer: We added the information of the construction to the manuscript's methods, as suggested. See below the referred topic at page 13 of the manuscript.

'Construction of the AS-*fmd* silenced *P. lutzii* strain

The *P. lutzii* formamidase gene (*fmd*) was silenced by the antisense RNA (aRNA) technique in conjunction with *Agrobacterium*-mediated transformation (ATMT), as previously described (2). Briefly, forward (5′ CCGCTCGAGCGGCTTGCATAACCGCTGGCATC 3′) and reverse (5′ GGCGCGCCTCGTCGGCGGAATCGTTATT 3′) oligonucleotides were designed to generate an antisense fragment (AS) of the *P. lutzii* formamidase gene. A binary plasmid (based on pUR5750) containing the AS-*fmd* and a gene for resistance to hygromycin B was then randomly inserted into the *P. lutzii* genome. Integration of this transfected cassette into the fungal genome was confirmed by conventional PCR using pUR5750 oligonucleotides.'

3. The authors discuss the following regarding the images in Figure 1: "The reduced intensity of dark-brown sites suggests a cellular response with lower melanogenesis compared to the wild-type (Fig. 1, J-L). These patterns of accumulation may also <u>indicate the presence of fungal melanin</u> (44), <u>which only became visually apparent due to the severe nodular melanin reduction</u>. Further analysis is needed to confirm this idea." What <u>specific analyses could be conducted to confirm this hypothesis</u>? It is important to explore this possibility further in the text.

Answer: We replaced these sentences to one making it clear that we did not perform any specific analysis to support an affirmation of this nature. See below the updated sentence (lines 2, 3, and 4 of page 5 from the manuscript).

'These patterns of accumulation could also indicate presence of fungal melanin (3), that requires additional investigation.'

4. The results presented in Figure 2 are insufficient to confirm the role of FMD as a DAMP during fungal-host interaction. This hypothesis needs to be explored in more detail. Are there other evidence that could support this specific role?

Answer: Thank you, your observation is correct. Indeed, our findings do not corroborate the hypothesis that formamidase plays a specific role in the process of immune recognition by the PRRs. In fact, the data actually indicate that this enzyme plays a role in the process of nodulation induction. However, further investigation is necessary to elucidate the underlying function and mechanism of it. Subsequently, the paragraph was revised to align its statements with the findings presented in this study. Please find the updated version below (page 8 of the manuscript, lines 3-to-10).

'Previous studies have described enzymatic activity playing a role of DAMP in *Drosophila* species (4). Moreover, PRRs are typically capable of recognizing a wide variety of microbial PAMPs, including peptides, glycans and saccharides (5). Here, we propose that the decrease in FMD at protein level notated in AS-*fmd* (2) along with the nodular changes discussed is a step towards incomplete nodulation(6). These findings suggest that formamidase may be involved in the process of triggering the immune response. Further investigation is required to elucidate the specific role of FMD in the generation of the immune response of *G. mellonella* though.'

5. The authors conclude that formamidase is associated with increased fungal burden in the nodules based on the results in Figure 3. Did the authors perform any statistical analysis to compare the groups? What strategies should be used to quantify the observed differences?

Answer: In the current illustration, our focus has not been on statistical analysis, but rather on an individual examination. The objective was to evaluate the collected specimens in order to identify any discrepancies in their tissue that could explain the significant reduction in mortality rates observed in the AS-*fmd*-infected larvae. Nevertheless, we recognize the necessity for further investigation into statistical issues. New studies on this subject could employ Colony Forming Units (CFU) or immunofluorescence assays to estimate the fungal load present in the insects' bodies.

6. The abstract highlights that the authors "suggest that formamidase is a crucial step in molecular recognition by *Galleria* immune cells." Please revise this sentence considering the points discussed above.

Answer: We replaced the recommended sentence with a proper one. Please, see it updated below.

'Thus, we suggest that formamidase may be a piece composing the process of molecular recognition by *Galleria* immune cells.'

Reviewer 2: Dear Editor, The manuscript by Pereira et. al. explores the participation of the *P. lutzii* formamidase in a model of *G. mellonella* infection by performing a series of histological evaluations and survival experiments comparing a wild type strain x silenced strain (AS-find) of *P. lutzii*. This manuscript is a nice contribution to the field, but some comments need to be addressed before its consideration for publication.

Here is a point-by-point comments for every section

ABSTRACT:

1. Page 5, line 8. The authors should re-write this sentence. What do they mean, the participation of formamidase or its products?

Answer: We revised the sentence as suggested.

INTRODUCTION

Some references are in numbered format and others (AUTHORS, year). The authors should always carefully review the whole document before submission. Also, the spaces between sentences and references.

Answer: You are definitely right. We revised the manuscript for space and references inconsistencies, as recommended.

1. What is the role of the ammonium generated by formamidase on the buffering of the pH? Please address.

Answer: The ammonium generated by amidases (or other virulence determinants/factors) plays a significant role in the pathogen's evasion strategy, specifically in the evasion of the phagosome. Ammonium's primary function of pH regulation is within the phagocytic cells, where an acidic environment is created with the intention of eradicating the microbe. In *Candida* species, ammonium extrusion is a crucial process that enables the morphological transition, as it is contingent upon maintaining a neutral pH (7).

2. Could the authors test the formamidase activity of these silenced strain (AS-find), in terms of cytoplasmic extracts and also exoantigens?

Answer: Thank you for your question. The enzymatic activity was part of the analysis and validation of the silenced mutants AS-*fmd*. Also, as shown by Silva and colleagues in 2022 (2), the expression of formamidase at protein level was reduced sixfold in the AS-*fmd* strain when compared to the wild-type. In addition, the AS-*fmd* strain also exhibited a decrease in formamidase specific activity (0.75 times) when compared to the wild type strain.

RESULTS AND DISCUSSION

1. Growth rate of both strains? Hygromycin plays any pressure on the AS-find strain?

Answwear: As previously described by Silva et al (2), the growth and viability of the strains were assessed with propidium iodide staining. The wild-type and AS-*fmd* strain were cultivated in BHI without hygromycin B, for a period of 96 hours. The optical density (OD) was measured on a daily basis at 600 nm in order to determine the growth curve. It was observed that the reduction of formamidase did not have a deleterious effect on fungal growth (as evidenced by the growth curve) or cell viability (as demonstrated by microscopy). Furthermore, the wild-type and AS-*fmd* strain were evaluated for mitotic stability in the absence or presence of hygromycin B. The transformed AS-*fmd* strain demonstrated the capacity to grow in the presence of hygromycin, whereas the wild-type strain exhibited impaired growth when this antibiotic was present.

2. Fig. 1. Could the authors measure the intensity of the spots and transform it to numeric data? Also, is the formation of these melanin spots correlated to the viability of the fungus? What happens if the authors inject a non-viable wild type to *G. mellonella*?

Answer: Thank you, for your contribution. Following reviewer 2 suggestions, we performed these analyses. Please find them in Page 6 'Reviewer 2 – Results' topic.

3. Could the authors perform any immunohistochemistry to accurately determine the arrangement of fungal cells in *G. mellonella* tissues upon infection?

Answer: To evaluate the arrangement of fungal cells, we elected to utilize the Gomori-Grocott silver methenamine staining method as an alternative to immunohistochemistry approach. As illustrated below, the fungal cells are distinguishable from the larval cells, thereby confirming the presence of HE structures.

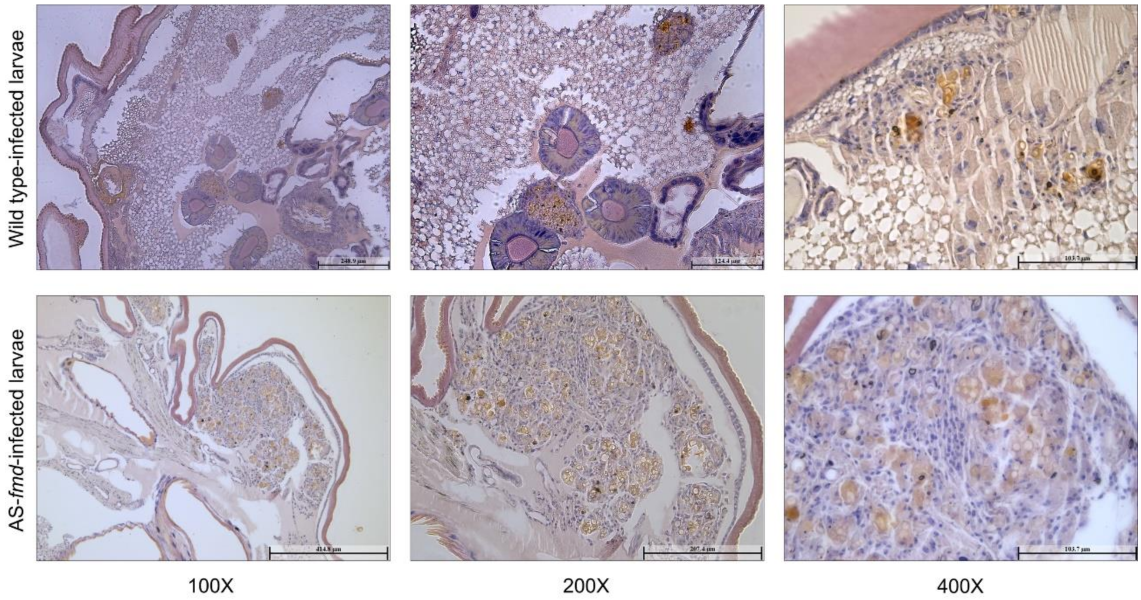

Figure 9 for reviewer' answers – Images from tissue slides of *G. mellonella* infected with wild type (top) and AS-*fmd* (bottom) *P. lutzii* strains. The images are magnified at 100X, 200X, and 400X, which evidences fungal cells in light brown.

4. It is not possible to accurately visualize the number of fungal cells comparing both strains (WT x AS-find silenced). Have the authors perform any CFU experiments to determine the viable fungal loads upon infection, as melanogenesis itself has been proven to have antimicrobial activities as the authors themselves stated.

Answer: Thank you for this observation. The histopathological data was assessed in order to correlate with the survival curve. In this work, no CFU experiments were conducted, and it is acknowledged that a quantitative evaluation of the viable fungal loads of each animal would be beneficial. Nevertheless, our objective was to provide a qualitative description of the circumstances surrounding the death of each animal.

5. What is the average of nodular tissues observed in WT x AS-fmd silenced infected *G. melonnella*?

Answer: The images displayed in Figure 3, which can be found on pages 2 and 3 of this response letter, were obtained in accordance with the instructions set forth in Figure 1 of this same document (page 1, Figure 1 for reviewer's answers). The images illustrate the qualitative characteristics of nodulation within the tissues of larvae infected with wild-type and AS-*fmd* strains. In this image, our objective was to evaluate four of the death events presented in the survival curve (Figure 4 of the manuscript). A quantitative analysis of the nodular counting would undoubtedly prove beneficial. Nevertheless, further studies will be conducted in conjunction with additional quantitative analyses and virulence traits of this model.

6. Could the authors include the data of phagocytosis and survival rates of WT x AS silenced strains upon interactions with haemocytes?

Answer: Due to unanticipated complications with the internalization analyses, we have elected to exclude these results from this manuscript. However, the methodology will be optimized for future evaluations.

7. What is the melanization pattern of the observed global larvae upon infection comparing both groups? Could the authors also perform a curve of melanization over time, for both groups and the controls?

Answer: Overall melanization of the larvae demonstrated slight alterations contingent on the designated groups. The data were not included in the article because the records are from a limited number of larvae and are of a qualitative nature, offering less insight than the histopathological analyses (Figure 10 for reviewers' answers). In this regard, no quantitative analysis or melanization curve was conducted on the experiments presented in this article. In order to demonstrate the global melanization of the larval groups, see the following images.

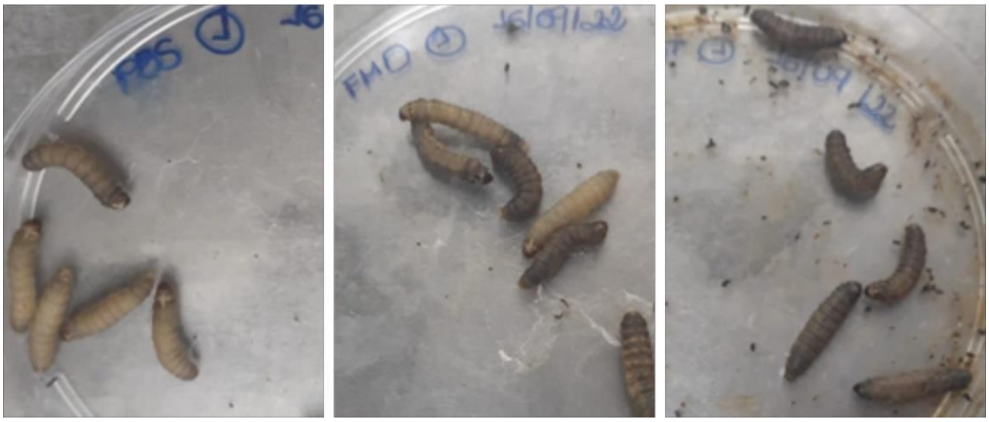

Figure 10 for reviewer' answers – The images illustrate the Galleria larvae at the seventh day post-infection. The initial image depicts the PBS-injected larvae, while the subsequent two images illustrate the AS-fmd and wildtype-infected specimens, respectively. The AS-*fmd* group presents clearer larvae than the wildtype. Additionally, some animals from AS-*fmd*-infected group exhibit a coloration pattern similar to that of the PBS-injected control group.

References used in this response letter

1. Silva LOS, Moreira TR, Gonçales RA, Tomazett MV, Parente-Rocha JA, Mattos K, et al. Paracoccidioides lutzii Formamidase Contributes to Fungal Survival in Macrophages. Microorganisms. 12 de outubro de 2022;10(10):2011.

2. Silva LOS, Moreira TR, Gonçales RA, Tomazett MV, Parente-rocha JA, Mattos K, et al. Paracoccidioides lutzii Formamidase Contributes to Fungal Survival in Macrophages. Microorganisms. 2022;10(10).

3. Smith DFQ, Casadevall A. The Role of Melanin in Fungal Pathogenesis for Animal Hosts. Em: Rodrigues ML, organizador. Fungal Physiology and Immunopathogenesis [Internet]. Cham: Springer International Publishing; 2019 [citado 15 de fevereiro de 2024]. p. 1–30. (Current Topics in Microbiology and Immunology; vol. 422). Disponível em: http://link.springer.com/10.1007/82_2019_173

4. Krautz R, Arefin B, Theopold U. Damage signals in the insect immune response. Front Plant Sci [Internet]. 11 de julho de 2014 [citado 22 de outubro de 2023];5. Disponível em: http://journal.frontiersin.org/article/10.3389/fpls.2014.00342/abstract

5. Dubovskiy IM, Kryukova NA, Glupov VV, Ratcliffe NA. Encapsulation and nodulation in insects. Invertebr Surviv J. 7 de julho de 2016;229-246 Pages.

6. Liu CT, Hou RF, Chen CC. Formation of basement membrane-like structure terminates the cellular encapsulation of microfilariae in the haemocoel of *Anopheles quadrimaculatus*. Parasitology. junho de 1998;116(6):511–8.

7. Hernández-Chávez MJ, Pérez-García LA, Niño-Vega GA, Mora-Montes HM. Fungal Strategies to Evade the Host Immune Recognition. J Fungi. 23 de setembro de 2017;3(4):51.

We would like to express our gratitude to the editor and reviewers for their valuable suggestions, which have proven to be essential in enhancing the quality of our manuscript. We look forward to hearing from you soon.

With best regards,

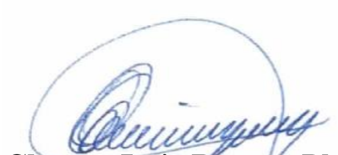

Clayton Luiz Borges, PhD

Laboratório de Biologia Molecular
Instituto de Ciências Biológicas
Universidade Federal de Goiás,
74690-900, Goiânia, Goiás, Brazil.
Tel:+55-62-3521-1110
e-mail:< clayton@ufg.bt>
